# RFID Data-Driven Vehicle Speed Prediction via Adaptive Extended Kalman Filter

**DOI:** 10.3390/s18092787

**Published:** 2018-08-24

**Authors:** Yupin Huang, Liping Qian, Anqi Feng, Yuan Wu, Wei Zhu

**Affiliations:** College of Information Engineering, Zhejiang University of Technology, Hangzhou 310023, China; 2111703090@zjut.edu.cn (Y.H.); 2111703044@zjut.edu.cn (A.F.); iewuy@zjut.edu.cn (Y.W.); weizhu@zjut.edu.cn (W.Z.)

**Keywords:** data acquisition, radio frequency identification, adaptive extended kalman filter, speed prediction

## Abstract

The traditional speed prediction generally utilizes the Global Position System (GPS) and video images, and thus, the prediction precision mainly depends on environmental factors (i.e., weather, ionosphere, troposphere, air, and electromagnetic waves). We study the Radio Frequency Identification (RFID) data-driven vehicle speed prediction and proposed an improved extended kalman filter (i.e., the adaptive extended kalman filter, AEKF) algorithm. Firstly, the on-board RFID reader equipped in the vehicle reads the information (i.e., current speed and time) from the tag deployed on the road. Secondly, the received information is transmitted to the on-board information processing unit, and it is demodulated and decoded into available information. Finally, based on the vehicle state space model, the AEKF algorithm is proposed to predict vehicle speed and improve the prediction results, so that the vehicle speed gradually approaches the actual vehicle speed. The simulation results show that compared with the conventional extended kalman filter (EKF) algorithm, our proposed AEKF algorithm improves the dynamic performance of the filtering and better suppresses the filtering divergence process. Moreover, the AEKF algorithm also improves the precision of the Mean Square Error (MSE) and Mean Absolute Error (MAE) by 57.4% and 32.4%, respectively.

## 1. Introduction

With the rapid development of social economy, urban road traffic jams and traffic accidents have become more and more common, and the traffic environment has become worse and worse [1,2]. To cope with these traffic problems, the concept of Intelligent Transportation System (ITS) has been proposed as a type of cutting-edge technology to improve the utilization of public transportation resources [3,4]. ITS is endowed with real-time monitoring, ubiquitous sensing, universal connectivity, intelligent information processing, and control [5,6,7]. To efficiently utilize the road resources and improve the driving safety, the vehicle speed predication is considered to be a promising research topic in the ITS [8,9,10]. Obviously, vehicle speed prediction can assist the drivers in adjusting their vehicle speed by referring to the speed of neighboring vehicles. Also, vehicle speed prediction can reduce the probability of various types of traffic accidents and improve the poor traffic environment. Therefore, it is of practical importance to accurately predict the speed of vehicles.

At present, road traffic speed prediction is mainly based on the geographic information from the Global Position System (GPS). GPS technology has been frequently applied to ITS with the implementation of Floating Car Data (FCD) [11]. In reference [12], Thiagarajan et al. proposed a Hidden-Markov-Model (HMM)-based map matching scheme that interpolates sparse data to identify the most probable road segments driven by the user. The OCTOTelematics system proposed in reference [13] used the real-time GPS data uploaded by 60,000 private vehicles to predict the real-time traffic speed based on the artificial network model and pattern matching model for Italian highways and major arterial roads in major citiesreference reference [14], Jenelius et al. segmented a road, calculated the most likely travelling time for each section based on the information of the driving trajectory of the floating car and finally, estimated the route travelling time. In reference [15], Zhang et al. established a K-Nearest Neighbor (KNN)-based short-term urban expressway traffic forecasting system from three aspects (i.e., historical database, search mechanism and algorithm parameters, prediction scheme). In [16], Gong et al. proposed a short-term traffic volume forecasting model based on Support Vector Regression (SVR), in which the traffic volumes in previous time periods were considered to be the input, and the traffic volume at the current time period was considered to be the output. Although the accuracy of speed measurement using GPS data is increasing, it still cannot change its own limitations. For example, GPS signals are blocked by obstacles when vehicles are driven to tunnels or overpasses, and they are also susceptible to environmental factors, such as under heavy tree canopies and in dense urban areas. Also, it is difficult to use GPS signals to predict the speed of neighboring vehicles via wireless communications due to the Doppler effect [17]. On the contrary, RFID tags are not affected by these environmental conditions, since RFID tags are generally waterproof, magnetically resistant, and resistant to high temperatures. To overcome the disadvantages of GPS signals, RFID technology can, therefore, be applied to vehicle speed prediction in the tunnel or under the overpass where the road surfaces can be prevented from significant abuse. In the literature, RFID technology has been used to predict vehicle speed [18,19]. For example, Algindy et al. proposed an average speed calculation method which calculates the vehicle speed by measuring the time required for a vehicle to pass two consecutive road markers deployed over a given distance [18]. In [19], Huo et al. proposed the weighted least squares method with a sliding window of 3 to calculate the weight of the velocity model and then estimated the vehicle speed. Although most efforts have been spent, the RFID data-driven vehicle speed prediction suffers low precision. To improve the prediction precision, we use the Kalman filter theory in this paper.

In terms of filtering algorithms, the Kalman filter is an algorithm that makes optimal use of imprecise data in a linear system with noises to continuously update the best estimate of the system’s current state [20]. In [21], Wu et al. developed a real-time object localization and tracking strategy based on the dynamic Kalman model for monocular image sequences in Unmanned Aerial Vehicle (UAV) navigation. In [22], Chen et al. proposed a Cubature Kalman-Extended target Probability Hypothesis Densit (CK-EPHD) filter to track targets in to non-linear Gaussian systems by combining the Cubature Kalman filter (CKF) with the Gaussian Mixtur-Extended target Probability Hypothesis Density (GM-EPHD) filter. In reference [23], Chen et al. proposed an Extended Kalman Filter (EKF)-based State Of Charge (SOC) estimation algorithm to estimate the charging states of batteries equipped in electric drive vehicles. In reference [24], Xia et al. proposed an adaptive fading Kalman filter algorithm for state estimationreference reference [25], Li et al. proposed a model-free method based on an adaptive Kalman filter to accomplish path tracking for a continuum robot. In reference [26], Chopin et al. proposed the Sequential Monte Carlo^2^ (SMC^2^) algorithm which propagates and resamples many particle filters in the x-dimension. In reference [27], Martino et al. designed an interacting parallel sequential Monte Carlo scheme for inference in state space models and online model selection.

This work aims to predict the current vehicle speed of the previous vehicle based on the speed information stored in the RFID tag. Therefore, we propose an RFID data-driven vehicle speed prediction algorithm based on adaptive extended kalman filter. The main contributions of this paper are summarized as follows:

- We propose an RFID-based vehicle speed predication system, in which the current vehicle can obtain the speed information of the previous vehicle via communication between the RFID tag and RFID reader.
- We design an improved EKF algorithm (e.g., AEKF) to improve the accuracy of vehicle speed prediction by combining the adaptive forgetting factor and the EKF algorithm.
- We design three vehicle speed models to simulate the driving environment. Meanwhile, we introduce two evaluation indicators (i.e., MSE and MAE) to better evaluate the vehicle speed prediction error.
- Extensive simulations are conducted to evaluate the advantages of the AEKF algorithm. The results show that the AEKF algorithm has less error than the conventional EKF algorithm in vehicle speed prediction.

The rest of this paper is organized as follows. In Section 2, we present the system model in detail. In Section 3, we provide some simulations to show that the effectiveness of our proposed road speed prediction. Finally, we summarize this paper in Section 4.

## 2. System Model

**Figure 1:**
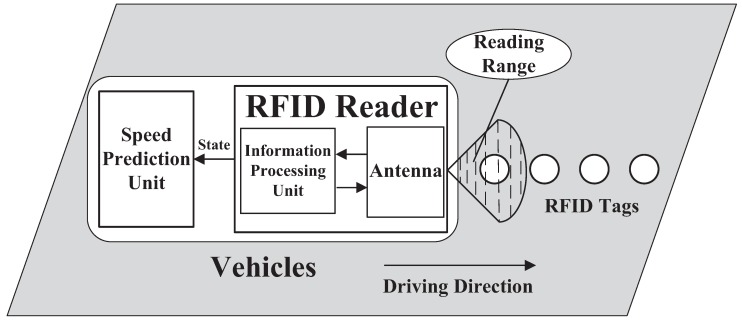
The diagram of RFID data-driven vehicle speed prediction system.

We considered a system model that mainly includes three parts: RFID tag deployment subsystem, information acquisition subsystem, and speed prediction subsystem. The system model is shown in Figure 1, and we firstly illustrate the RFID tag deployment subsystem in the next subsection.

### 2.1. RFID Tag Deployment Subsystem

The RFID tag deployment subsystem shown in Figure 1 consists of a RFID tag deployed on the road and an antenna in a vehicle reader. We next explain it in detail.

First of all, RFID tags were divided into three types (i.e., passive, active, and semi-passive tags) depending on whether or not the internal power supply is equipped. For RFID deployment into roads, we used passive RFID tags which have the advantages of small size, light weight, low cost, long life, and long maintenance period. Meanwhile, the passive tags supported Tag Talks First (TTF). The RFID tag was deployed on the lane center line and adopted an equidistant deployment strategy. The vehicle speed corresponded to the dashboard speed obtained with the inertial sensors equipped in the vehicle. The RFID tag was only used to store vehicle status information (e.g., the vehicle’s current speed and the current time). In addition, considering the limitations of the storage capacity of the RFID tag, the tag only stored the latest vehicle status information.

Secondly, in terms of vehicle reader, the reader was installed in the center of the front bumper of the vehicle, and the antenna of the reader was at an angle to the horizontal plane to ensure that the tag could be identified. A reader’s reading range depends on many physical and geometric parameters. The specific equation is as follows [28]:

(1) $$\begin{array}{c} P_{\mathrm{RX},\mathrm{tag}}=P_{\mathrm{TX},\mathrm{reader}}T_{b}G_{\mathrm{reader}}G_{\mathrm{tag}}{\left(\frac{\lambda }{4\pi r}\right)}^{2} \end{array}$$

(2) $$\begin{array}{c} P_{\mathrm{RX},\mathrm{reader}}=P_{\mathrm{TX},\mathrm{reader}}T_{b}G_{\mathrm{reader}}^{2}G_{\mathrm{tag}}^{2}{\left(\frac{\lambda }{4\pi r}\right)}^{4} \end{array}$$

Here, $P_{\mathrm{RX},\mathrm{tag}}$ denotes the received power of the RFID tag, $P_{\mathrm{RX},\mathrm{reader}}$ denotes the reader’s received power, $P_{\mathrm{TX},\mathrm{reader}}$ denotes the transmitted power of the reader, $T_{b}$ denotes the backscattered transmission loss, $G_{\mathrm{reader}}$ denotes the reader’s transmitted antenna gain, $G_{\mathrm{tag}}$ denotes the gain of the tag antenna, *r* denotes the distance between the RFID tag and the reader, and λ denotes the wavelength.

**Figure 2:**
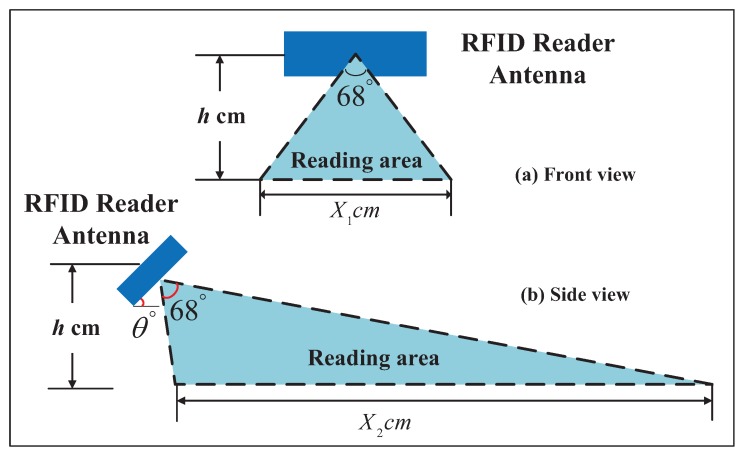
The reading area of RFID reader.

According to Equation (1) and Equation (2), the reading range is mainly affected by the reader transmit power and the antenna installation angle. In reference [29], Chon et al. pointed out that the RFID reader’s antenna center was used as a fixed point, and the conical region with a radiation angle of $68^{\circ }$ was used as the read/write area of the reader. On the basis of this, a corresponding calculation method of the readability of the reader antenna in the road surface area is given in Figure 2. Here, we have the length and the width of the reading area (denoted by $X_{1}$ and $X_{2}$, respectively), such that

(3) $$\begin{array}{c} X_{1}=2\times h\times \tan34^{\circ } \end{array}$$

(4) $$\begin{array}{c} X_{2}=\frac{h}{\tan(56^{\circ }+\theta^{\circ })}+\frac{h}{\tan(56^{\circ }-\theta^{\circ })}, \end{array}$$

where $\theta^{\circ }(-56^{\circ }<\theta^{\circ }<56^{\circ })$ denotes the angle between the antenna and the horizontal direction, and *h* denotes the height between the center of the RFID reader antenna and the road surface. For example, when $\theta^{\circ }=45^{\circ }$ and h=37.5 cm, we can calculate the values of $X_{1}$ and $X_{2}$ as 58.58 cm and 185.63 cm, respectively.

Considering that the tags are too densely paved, readers in RFID systems may experience tag information collisions (i.e., reading two or more tags’ information at the same time). In order to solve this problem effectively, it is necessary to ensure that the distance between the tag and the tag is sufficiently large. Therefore, the design criteria of our road system are explained as follows:

1. The read area of each RFID reader should cover no more than one tag at any moment.
2. Each RFID tag should be covered by no more than one RFID reader’s read area at any moment.
3. The premise that the vehicle can read the tags deployed in the lane is that the vehicle is in the lane.
4. If a vehicle can read a tag, at least half of its body should be in the lane where the tag is deployed.
5. If less than half of a vehicle is in a lane, the vehicle cannot read any tags’ information in the lane.
6. We set $\theta^{\circ }=45^{\circ }$ and *h* = 37.5 cm, and thus, the distance between tags should be at least 185.63 cm.

After illustrating the RFID tag deployment subsystem, we illustrate the information acquisition subsystem in the next subsection.

### 2.2. Information Acquisition Subsystem

The information acquisition subsystem exists in the reader. The RFID tag information is received by the reader antenna, then sent to the information processing unit and demodulated and decoded into usable information.

Specifically, the reader Radio Frequency (RF) antenna emits electromagnetic waves of a certain frequency and forms an effective reading area around the bumper of the vehicle. As the vehicle travels, the RFID tags enter the reading area, and the tags begin to gain energy due to the inductive coupling between the reader and the tags. The built-in rectification circuit of the tag rectifies and smooth the energy to generate a direct voltage. When the direct voltage reaches the required operating voltage of the circuit, the circuit in the tag is activated. Firstly, the tag can transmit the information to the reader in a load-modulated manner. After receiving the modulation signal, the reader antenna sends the modulation information to the information processing unit and then demodulates and decodes it into usable information. In addition, when the antenna receives the signal, the dashboard speed obtained via the inertial sensors equipped in the vehicle should be sent to the RFID tag via the RFID reader. In addition, the current state information of the vehicle will be transmitted to the reader antenna after being coded and modulated by the information processing unit. Afterwards, the antenna is written to the tag via the 24-bit EPC (Electronic Product Code) code to replace the original status information in the tag.

Considering that the time between passing two tags is very short when the vehicle is driving at a high speed, and the speed usually gradually changes in different environments (i.e., tunnel), we only needed to collect the historical information of the vehicle speed to predict a vehicle’s speed. In this paper, we describe the state equation and observation equation of the vehicle speed in our system model as follows:

(5) $$\begin{array}{c} x_{k+1}=f\left(x_{k}\right)+W_{k} \end{array}$$

(6) $$\begin{array}{c} z_{k}=h\left(x_{k}\right)+V_{k} \end{array}$$

where $x_{k}$ denotes the state vector of the system, and $z_{k}$ denotes the noisy observation vector of the system. The random variables $W_{k}$ and $V_{k}$ denote the process noise and measurement noise, respectively. We assume that $W_{k}$ and $V_{k}$ are white noises that are independent of each other and with a Gaussian distribution:

(7) $$\begin{array}{c} W_{k}∼N\left(0,Q_{k}\right) \end{array}$$

(8) $$\begin{array}{c} V_{k}∼N\left(0,R_{k}\right) \end{array}$$

where $Q_{k}$ denotes the process noise covariance, and $R_{k}$ denotes the measurement noise covariance.

In the next subsection, we focus on illustrating the speed prediction subsystem.

### 2.3. Speed Prediction Subsystem

The speed prediction subsystem is based on RFID data and integrated with the vehicle speed state space model. It adopts the AEKF algorithm to realize the vehicle speed prediction. The AEKF algorithm is mainly divided into four parts: linearization, time update process, measurement update process, and adaptive forgetting factor. In the following text, we present the details of the AEKF algorithm, in which the key ideas come from the conventional EKF algorithm.

- **Linearization**

The AEKF algorithm transforms nonlinear problems into linear problems by linearization. In Taylor’s expansion, we can perform second-order truncation, and even third-order truncation and fourth-order truncation. On the one hand, doing this can reduce the estimation error caused by linearization and slightly improves the estimation accuracy. On the other hand, this greatly increases the number of iterations of data, and it is difficult to implement online. The details of Taylor’s expansion are as follows:

(9) $$\begin{array}{c} f\left(x_{k}\right)=f\left({\hat{x}}_{k}\right)+\phi_{k+1|k}\left(x_{k}-{\hat{x}}_{k}\right)+\Delta t_{1} \end{array}$$

(10) $$\begin{array}{c} \phi_{k+1|k}=\frac{\delta f({\hat{x}}_{k})}{\delta {\hat{x}}_{k}} \end{array}$$

(11) $$\begin{array}{c} h\left(x_{k}\right)=h\left({\hat{x}}_{k}\right)+H_{k}\left(x_{k}-{\hat{x}}_{k}\right)+\Delta t_{2} \end{array}$$

(12) $$\begin{array}{c} H_{k}=\frac{\delta h({\hat{x}}_{k})}{\delta {\hat{x}}_{k}} \end{array}$$

where $\phi_{k+1|k}$ denotes the system state transition matrix which relates the state at the previous time step *k* to the state at the current step k+1. In addition, $H_{k}$ denotes the observation matrix which relates the state to the measurement ($z_{k}$). $\Delta t_{1}$ and $\Delta t_{2}$ are infinitesimal above the first order. Ignoring higher-order infinitesimals, substituting Equation (9) into Equation (5) and substituting Equation (11) into Equation (6) gives

(13) $$\begin{array}{c} x_{k+1}=f\left({\hat{x}}_{k}\right)+\phi_{k+1|k}\left(x_{k}-{\hat{x}}_{k}\right)+W_{k} \end{array}$$

(14) $$\begin{array}{c} z_{k}=h\left({\hat{x}}_{k}\right)+H_{k}\left(x_{k}-{\hat{x}}_{k}\right)+V_{k} \end{array}$$

- **Time Update Process**

The time update process firstly obtains the a priori estimate of the next time step based on the current state. Then the a priori error covariance is calculated with the adaptive forgetting factor (μ). In addition, the time update process can also be thought of as a prediction process.

(15) $${\hat{x}}_{k+1|k}=f\left({\hat{x}}_{k|k}\right)$$

We defined ${\hat{x}}_{k+1|k}$ to be our a priori estimate at step k+1 from the previous prediction, and ${\hat{x}}_{k|k}$ to be the a posteriori state estimate at step *k*. The a priori estimate error covariance matrix ($P_{k+1|k}$) is presented in Equation (16):

(16) $$P_{k+1|k}=\mu_{k}\phi_{k+1|k}P_{k|k}\phi_{k+1|k}^{T}+Q_{k+1}$$

Among them, the adaptive forgetting factor ($\mu_{k}$) can increase the weight of the latest data and reduce the weight of historical data. At the same time, $\mu_{k}$ also corrects the a priori estimate error covariance ($P_{k+1|k}$).

- **Measurement Update Process**

The measurement update process is responsible for the correction, i.e., for incorporating a new measurement into the a priori estimate to obtain an improved a posteriori estimate. In addition, the measurement update process can be thought as a correction process.

The posteriori state estimate (${\hat{x}}_{k+1|k+1}$) is presented in Equation (17):

(17) $${\hat{x}}_{k+1|k+1}={\hat{x}}_{k+1|k}+K_{k+1}\left\{z_{k+1}-h\left({\hat{x}}_{k+1|k}\right)\right\}$$

where $h({\hat{x}}_{k+1|k})$ denotes a presumption of the pre-measurement value. We hope that the filter can filter out noise that interferes with the actual state, i.e., the estimated state of the filter is closest to the actual state. The closest one can be understood as the smallest two-norm sum of errors between the actual state and the estimated state at step *k*, which is equivalent to the trace minimum of the covariance matrix, as shown in Equation (18):

(18) $$\min_{K_{k+1}}\sum {∥x_{k+1}-{\hat{x}}_{k+1|k+1}∥}_{2}^{2}⟺\min_{K_{k+1}}tr\left\{cov(x_{k+1}-{\hat{x}}_{k+1|k+1})\right\}=\min_{K_{k+1}}tr\left(P_{k+1|k+1}\right)$$

The a posteriori estimate covariance matrix ($P_{k+1|k+1}$) is presented in Equation (19):

(19) $$\begin{array}{cc} P_{k+1|k+1} & =cov(x_{k+1}-{\hat{x}}_{k+1|k+1})\\ & =cov\left\{\left(I-K_{k+1}H_{k+1}\right)\left(x_{k+1}-{\hat{x}}_{k+1|k+1}\right)\right\}+K_{k+1}R_{k+1}K_{k+1}^{T}\\ & P_{k+1|k}\left(I-H_{k+1}^{T}K_{k+1}^{T}\right)+K_{k+1}\left\{\left(H_{k+1}P_{k+1|k}H_{k+1}^{T}+R_{k+1}\right)K_{k+1}^{T}-H_{k+1}P_{k+1|k}\right\} \end{array}$$

The above expected value corresponds to minimizing the trajectory of the a posterior estimate covariance matrix. In Equation (20), the trace is minimized when the matrix derivative is zero. Solving Equation (20) yields the optimal Kalman gain ($K_{k+1}$), as shown in Equation (21):

(20) $$\begin{array}{c} \frac{\delta P_{k+1|k+1}}{\delta K_{k+1}}=2H_{k+1}P_{k+1|k+1}H_{k+1}^{T}K_{k+1}^{T}+2R_{k+1}K_{k+1}^{T}-{\left(P_{k+1|k}H_{k+1}^{T}\right)}^{T}-H_{k+1}P_{k+1|k}^{T}=0 \end{array}$$

(21) $$\begin{array}{c} K_{k+1}=P_{k+1|k}H_{k+1}^{T}{\left(H_{k+1}P_{k+1|k}^{T}H_{k+1}^{T}+R_{k+1}\right)}^{-1} \end{array}$$

where $P_{k+1|k}$ and $H_{k+1}P_{k+1|k}^{T}H_{k+1}^{T}+R_{k+1}$ are symmetric matrices, and the kalman gain ($K_{k+1}$) is selected to minimize the a posteriori state estimate by incorporating the measurement. Substituting Equation (21) into Equation (19) in order to simplify $P_{k+1|k+1}$ gives

(22) $$P_{k+1|k+1}=\left(I-K_{k+1}H_{k+1}\right)P_{k+1|k}$$

- **Adaptive Forgetting Factor**

We know that as $\mu_{k}$ increases, this eventually leads to ${\hat{x}}_{k+1|k+1}$ getting closer to the observed value (e.g., the dynamic performance of the system increases, and the error also greatly increased). Meanwhile, the value of the adaptive forgetting factor ($\mu_{k}$) will change as the system model parameters change, thus ensuring the tracking accuracy of the system model. In addition, the calculation of the adaptive forgetting factor when k>0 is as follows:

(23) $$\begin{array}{c} \mu_{k}=max\left\{1,\alpha \frac{tr(G_{k})}{tr(\xi_{k})}\right\} \end{array}$$

(24) $$\begin{array}{c} G_{k}=d\left(M_{k}-H_{k}Q_{k}H_{k}^{T}-R_{k}\right) \end{array}$$

(25) $$\begin{array}{c} e_{k}=z_{k}-h\left({\hat{x}}_{k|k-1}\right) \end{array}$$

(26) $$\begin{array}{c} d=\left\{\begin{array}{cc} \frac{U}{e_{k}e_{k}^{T}}, & \mathrm{if}\;e_{k}e_{k}^{T}\geq U\\ 1, & \mathrm{if}\;e_{k}e_{k}^{T}<U \end{array}\right. \end{array}$$

(27) $$\begin{array}{c} M_{k}=\frac{\mu_{k-1}e_{k}e_{k}^{T}}{1+\mu_{k-1}} \end{array}$$

(28) $$\begin{array}{c} \xi_{k}=H_{k}\phi_{k|k-1}P_{k-1|k-1}\phi_{k|k-1}^{T}H_{k}^{T} \end{array}$$

where α (α>1) denotes a correction coefficient which can coercively improve the tracking performance of the filter, and the value of α depends on the actual situations. *U* denotes the tolerable maximum error variance, and the smaller the value of *U* is, the higher the accuracy requirement is. Meanwhile, the value of *U* depends on the actual situations. $e_{k}$ denotes the residual, which is the difference between the actual measured value and the estimated output value at step *k*, and $e_{k}$ reflects the tracking ability of the system state. Furthermore, the smaller the value of $e_{k}$, the stronger the tracking ability is. $M_{k}$ is the covariance matrix of the residual $e_{k}$. *d* denotes the weighting factor, which can affect $G_{k}$ and then affect $\mu_{k}$. In Equation (23), $\mu_{k}$ depends, to a large extent, on α and $G_{k}$, and $G_{k}$ is affected by *d*. Therefore, when the residual exceeds the maximum tolerable error, we must reduce the value of *d* to ensure the system’s accuracy. When the residual error is no more than the maximum tolerable error, we tend to improve the dynamic characteristics of the system, and we thus set d=1. In Equation (27), we can see that $M_{k}$ directly uses the information of the current moment instead of averaging the historical information. In addition, it can reflect the status quo of the system model error at the current moment. $G_{k}$ and $\xi_{k}$ are intermediate variables with no specific physical meaning. Considering that there is an error between the established model and the actual situation, the accumulated error will continue to be accumulated in the filtering calculation process. This causes $P_{k+1|k}$ to lose its positive definite symmetry, making the filter’s dynamic tracking performance weak and even causing the filter to diverge. The purpose of introducing the forgetting factor is to limit the memory length of the filter by the forgetting factor, make full use of the latest measurement data, improve the dynamic performance of the system, and improve the tracking effect of the filter. Specifically, it can be seen from Equation (23) to Equation (28) that when the vehicle state is abrupt, the increase of the residual will lead to an increase in the error variance matrix, and the forgetting factor will increase accordingly. In addition, we set the value of $\mu_{0}$ to 1. When $\mu_{k}>1$, this indicates that the system needs to improve the a priori covariance matrix to ensure that the estimated value can track the latest observations. When $\mu_{k}$ = 1, it is consistent with the conventional EKF algorithm.

**Figure 3:**
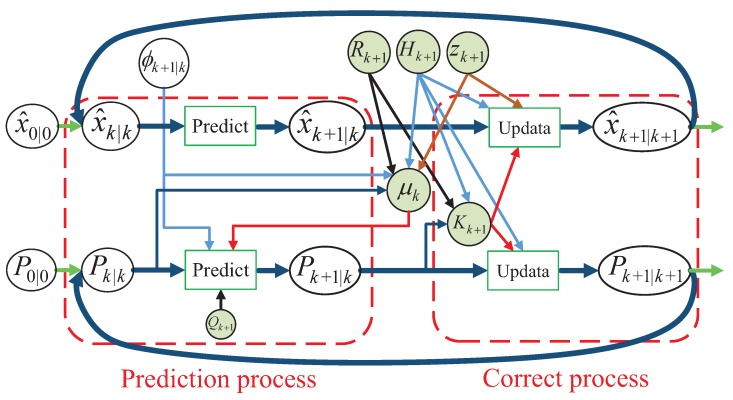
The operation of the AEKF algorithm.

In addition, the complete operation of the AEKF algorithm is described in Figure 3. The adaptive extended kalman filter estimates the process by using a form of feedback control, i.e., the filter obtains feedback while estimating the state of the process. In addition, the design of an RFID data-driven vehicle speed prediction based on an adaptive extended kalman filter is specifically as shown in Algorithm 1:

**Algorithm 1.** RFID data-driven speed prediction based on the AEKF algorithm.
1:**Initialization:** Set k=0 and initialization variables (i.e., $\phi_{k+1|k}$, $P_{k+1|k}$, $H_{k+1}$, $\mu_{k}$, α, *d*)2:**while** the vehicle is driving in the RFID road system **do**3:  Collect the information in the k+1 RFID tag.4:  Calculate $\phi_{k+1|k}$ and $H_{k}$.5:  **if** k > 0 **then**6:   Calculate ${\hat{x}}_{k+1|k}$ and $e_{k}$ according to the Equation (15) and Equation (25), respectively.7:   **if** $e_{k}$$e_{k}^{T}$ < *U* **then**8:    Set d=1.9:   **else**10:    Set $d=\frac{U}{e_{k}e_{k}^{T}}$.11:   **end if**12:   Calculate $G_{k}$ and $\xi_{k}$ according to the Equations (24) ∼ (28), respectively.13:   Calculate $\mu_{k}$ according to the Equation (23).14:   Calculate $P_{k+1|k}$, ${\hat{x}}_{k+1|x+1}$, $K_{k+1}$ and $P_{k+1|k+1}$ according to the Equations (16), (17), (21), (22), respectively.15:  **else**16:   This information is used as the initial value of the system, and set $\mu_{k}$=0.17:  **end if**18:  Set k=k+119:
**end while**



## 3. Simulation Analysis

We used MATLAB for simulation experiments. We firstly introduced two evaluation indicators and established three vehicle models to verify the effectiveness of our proposed RFID data-driven vehicle speed prediction.

### 3.1. Evaluation Indicator Setting

We firstly introduced the MSE as one of the evaluation indicators for vehicle speed tracking prediction to evaluate the degree of change in the vehicle speed. The smaller the value of MSE is, the more accurately a prediction model describes the experimental data. The MSE is defined as follows:

(29) $$MSE=\frac{1}{N}\sum_{k=1}^{N}{\left\{X_{a}\left(k\right)-X_{p}\left(k\right)\right\}}^{2}$$

where *N* denotes the number of simulations. *k* denotes the *k*-th simulation. $X_{a}\left(k\right)$ denotes the *k*-th actual value. $X_{p}\left(k\right)$ denotes the *k*-th prediction. We then introduced the MAE to better reflect the actual situation of the prediction error. The MAE is defined as follows:

(30) $$MAE=\frac{1}{N}\sum_{k=1}^{N}\left|X_{a}\left(k\right)-X_{p}\left(k\right)\right|$$

The parameter definitions were consistent with the MSE.

### 3.2. Simulation Model Setting

Assuming the vehicle is traveling in the tunnel, we set the initial position of the vehicle to zero. Meanwhile, we also assumed that the length of the tunnel was 4 km. Four hundred and one tags were deployed on the road with a tag spacing of 10 m, and RFID tags were also deployed at the initial position. In addition, the values of $Q_{k}$ and $R_{k}$ were set to 1 and 2, respectively. In addition, we set *d* and *U* to 1 and 0.5, respectively. We established three speed models, as follows:

- Normal model: Vehicle moves with a constant speed at 25 m/s for 30 s, then it decelerates by −0.3 m/s^2^ for 50 s. After reaching 10 m/s, the vehicle accelerates at 0.4 m/s^2^ for 50 s. Finally, the vehicle moves with a constant speed of 25 m/s for 30 s.
- Constant speed model: Vehicle moves with a constant speed at 25 m/s for 100 s.
- Deceleration model: Vehicle moves with a constant speed at 25 m/s for 4 s, and then it decelerates at −2.5 m/s^2^.

### 3.3. Analysis of Simulation Results

**Figure 4:**
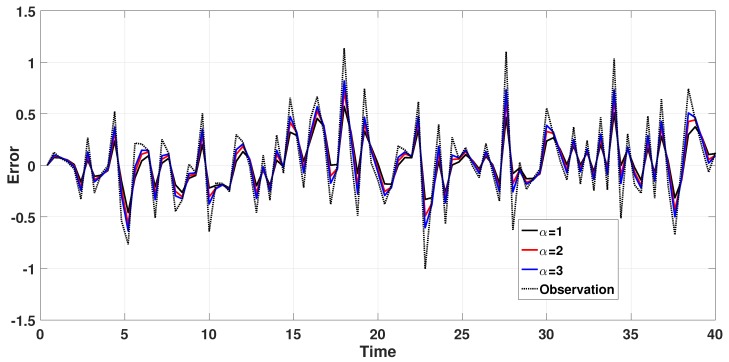
Errors with different α values.

First of all, we discussed the effects of different values of the correction coefficient α on the system’s identification accuracy when the vehicle is moving at a constant speed at an initial speed. In addition, we randomly generated $Q_{k}$ and $R_{k}$ that obey a Gaussian distribution and record these. We set the number of iterations to 100 and used the same $Q_{k}$ and $R_{k}$ in each simulation to ensure that α was the only independent variable. We then took the value of α from 1 to 3, and the step size was set to 0.1. The simulation results are shown in Figure 4 and Table 1.

When the system state variable mutates, it quickly accumulates errors and makes the filter’s dynamic tracking performance weaker, and it may even diverge. The adaptive forgetting factor limits the memory length of the filter and makes full use of the latest measurement. At the same time, the correction coefficient (α) can increase the adaptive forgetting factor and improve the system’s dynamic performance. In addition, dynamic performance is described as the ability to track observations. As shown in Figure 4, we selected three typical values of α. As the correction coefficient (α) increased, the error kept increasing and the predictions became closer and closer to the observations. The reason for is that the introduction of α made the optimal filtering into sub-optimal filtering at the expense of a certain degree of filtering accuracy for better system dynamic performance. As shown in Table 1, it can be seen that as the value of α increases, the rate of increase of MSE increases with the increase of α, and the rate of increase of MAE decreases with the increase of α. In summary, in order to balance the dynamic characteristics and errors of the system, we set the value of α = 2 to 2.

**Figure 5:**
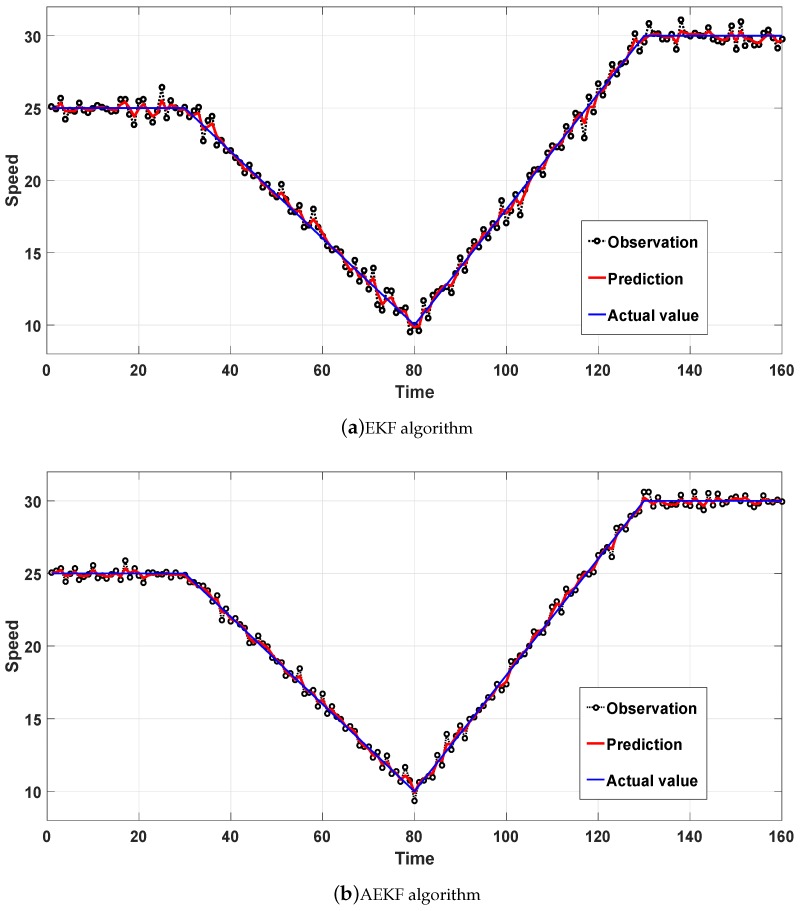
Speed prediction effect in the normal model.

**Figure 6:**
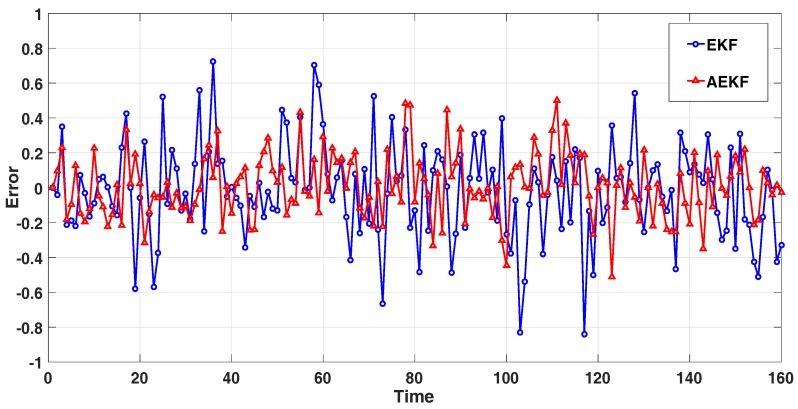
Speed error effect in the normal model.

We then applied the EKF algorithm and the AEKF algorithm to the vehicle speed normal model. We divided the normal model into four stages, which are constant speed, deceleration, acceleration, and constant speed in that order. The predicted effects of the EKF and AEKF algorithms are shown in Figure 5. In addition, the error comparison of the two algorithms is shown in Figure 6.

In Figure 5, the lines represent the observation, prediction, and actual value, respectively. The closer the predicted value is to the actual value, the better the prediction effect of the system is. In Figure 5a, the maximum error at the 117th second is 0.8399 m/s. In Figure 5b, the maximum error of 0.5122 m/s occurred at the 123th second. In Figure 6, we can directly see that the filtering accuracy of the AEKF algorithm is higher than the EKF algorithm, which indicates that the AEKF algorithm can better predict the speed of high-speed moving vehicles. We also divided four stages of the normal model for comparison, and the error comparisons of the two algorithms are shown in Table 2.

In Table 2, we can see that the accuracy improvement of the AEKF algorithm relative to the EKF algorithm in the MSE are 61.8%, 57.0%, 51.5%, and 60.6%, respectively. In terms of MAE, the accuracy improvement of the AEKF algorithm relative to the EKF algorithm is 31.4%, 29.3%, 25.5%, and 38.9%, respectively. In general, the AEKF algorithm improves MSE and MAE by 56.3% and 30.1%, respectively, over the EKF algorithm.

**Figure 7:**
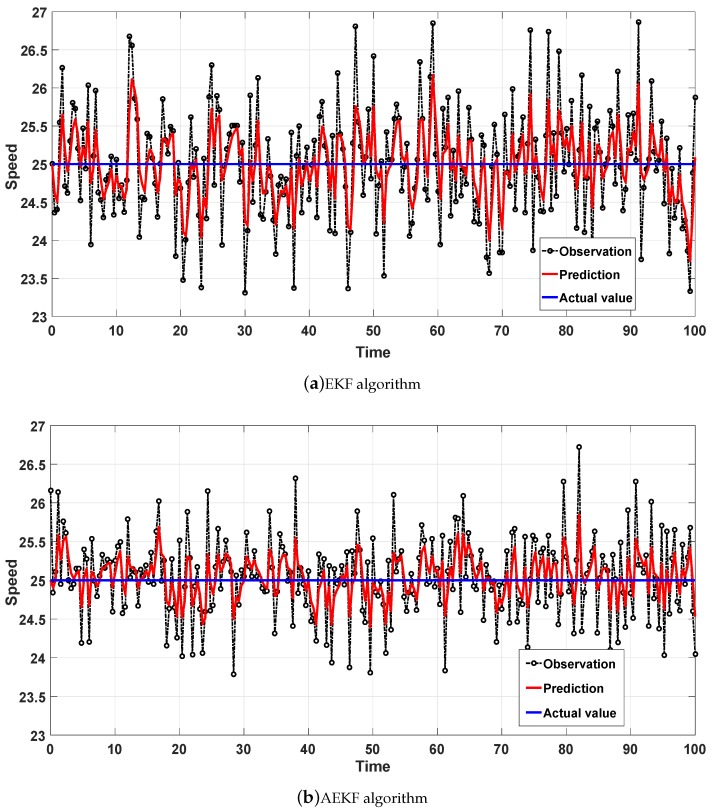
Speed prediction effect in the constant speed model.

We then applied the EKF algorithm and the AEKF algorithm to the constant speed model. The simulation results are shown in Figure 7.

First, constant speed mode is often used when the vehicle is driving on the highway. We can see that velocity predicted value is in the range of 24 to 26 with the EKF algorithm in Figure 7a. Meanwhile, we also can see that the velocity prediction value is in the range of 24.5 to 25.5 with the AEKF algorithm in Figure 7. In addition, an increase in vehicle travelling time (e.g., the number of iterations in each simulation) in constant speed model results in an increase in the error at the same number of simulations, but the increase in accuracy is less affected. In addition, the error comparisons in the constant speed model are shown in Table 3.

**Figure 8:**
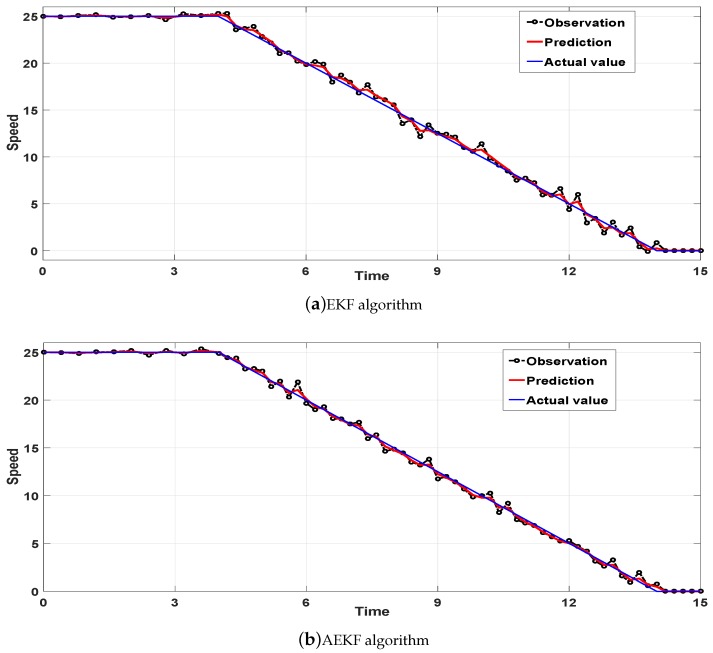
Speed prediction effect in the deceleration model.

The speed prediction effect of the EKF and AEKF algorithms in deceleration model is shown in Figure 8a. Figure 8b shows that the AEKF algorithm achieves a smaller error than the EKF algorithm. In Table 3, the precision improvemenst of the three models are shown to be above 50% and 30%. Among them, the AEKF algorithm has the highest accuracy improvement in the constant speed model. In detail, the AEKF algorithm improved the MSE aspects of the three modes with accuracy levels of 56.3%, 59.6%, and 54.5%, respectively. In addition, the AEKF algorithm improved the MAE aspects of the three modes with accuracy levels of 30.1%, 35.3%, and 30.4%, respectively. Finally, we calculated the average error of the three vehicle speed models and found that the algorithm accuracy increases were 57.4% and 32.4%. Therefore, it is concluded that under the same initial conditions, the AEKF algorithm’s vehicle speed prediction errors are smaller than those of the EKF algorithm.

## 4. Conclusions

To account for the phenomenon in which GPS signals cannot work normally because of the environment (i.e., tunnel), we proposed an RFID road system based on the AEKF algorithm to predict the speed of high-speed moving vehicles. In Section 3, we firstly discussed the influence of different correction coefficients α on the system under conditions in which the initial conditions were unchanged. We found that with an increase in the correction coefficient (α), the dynamic performance of the system greatly improved, but the filtering accuracy was lost. We then compared the AEKF algorithm with the EKF algorithm in three modes. The mean square error and the mean absolute error of the AEKF algorithm were both smaller than those of the EKF algorithm. The AEKF algorithm had a 57.4% and 32.4% lower mean square error and relative absolute error than the EKF algorithm, respetively. At the same time, the system can still quickly and effectively predict the target speed while saving costs, ensuring the safety of the vehicle and reducing the probability of a traffic accident.

## Figures and Tables

**Table 1:** Comparison with different α values.

α	1	1.2	1.4	1.6	1.8	2	2.2	2.4	2.6	2.8	3
**MSE**	0.0396	0.0441	0.0485	0.0530	0.0572	0.0613	0.0653	0.0690	0.0724	0.0757	0.0788
**MAE**	0.1525	0.1614	0.1702	0.1784	0.1861	0.1931	0.1994	0.2052	0.2105	0.2152	0.2195

**Table 2:** Algorithmic errors in the normal model.

Stage	EKF		AEKF		EKF vs AEKF	
	MSE	MAE	MSE	MAE	MSE	MAE
**1**	0.0608	0.1833	0.0232	0.1257	61.8%	31.4%
**2**	0.0828	0.2114	0.0356	0.1494	57.0%	29.3%
**3**	0.0862	0.2247	0.0418	0.1673	51.5%	25.5%
**4**	0.0609	0.2017	0.0240	0.1233	60.6%	38.9%
**Total**	0.0756	0.2084	0.0330	0.1457	56.3%	30.1%

**Table 3:** Algorithmic errors in the vehicle speed model.

Stage	EKF		AEKF		EKF vs AEKF	
	MSE	MAE	MSE	MAE	MSE	MAE
**Normal model**	0.0756	0.2084	0.0330	0.1457	56.3%	30.1%
**Constant speed model**	0.1746	0.3269	0.0705	0.2115	59.6%	35.3%
**Deceleration model**	0.1085	0.2489	0.0493	0.1732	54.5%	30.4%
**Average**	0.1196	0.2614	0.0509	0.1768	57.4%	32.4%
